# Formation of porous SnS nanoplate networks from solution and their application in hybrid solar cells†
†Electronic supplementary information (ESI) available: Details to performed experiments and characterisation methods, additional XRD data, absorption spectra, TAS data and SEM images. See DOI: 10.1039/c5cc03125g



**DOI:** 10.1039/c5cc03125g

**Published:** 2015-05-27

**Authors:** T. Rath, L. Gury, I. Sánchez-Molina, L. Martínez, S. A. Haque

**Affiliations:** a Department of Chemistry and Centre for Plastic Electronics , Imperial College London , Imperial College Road , London , SW7 2AZ , UK . Email: t.rath@imperial.ac.uk ; Email: s.a.haque@imperial.ac.uk

## Abstract

Herein, we present a facile solution-based route towards nanostructured, hybrid absorber layers based on tin mono-sulfide (SnS), an emerging, non-toxic absorber material for low-cost and large-scale PV applications. Charge photogeneration properties in the hybrid system are studied using transient absorption spectroscopy and fabricated solar cells show efficient photocurrent generation over a broad spectral range.

## Abstract 2

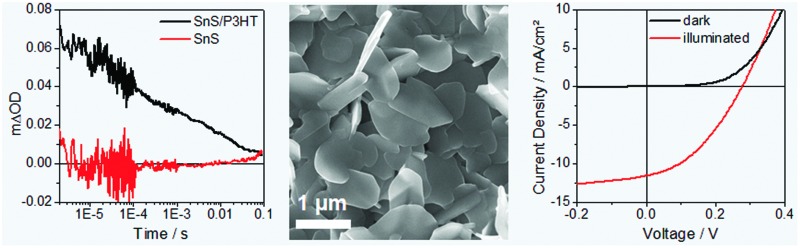
Herein, we present a facile solution-based route towards nanostructured, hybrid absorber layers based on tin mono-sulfide (SnS), an emerging, non-toxic absorber material for low-cost and large-scale PV applications.

The aim of preparing low-cost solar cells with high power conversion efficiency in large-scale using sustainable, non-toxic materials motivated researchers in the last years to develop and investigate materials capable of reaching this target. The most prominent example within this class of materials is copper zinc tin sulfide (CZTS) to which much research effort was directed and remarkable progress was made in recent years.^1,2^ Another material that is currently generating interest for photovoltaic applications is tin mono-sulfide (SnS). SnS is a non-toxic material consisting of abundant and cheap elements, which possesses a high absorption coefficient (>10^4^ cm^–1^) and a band gap (1.3 eV) optimal for photovoltaic applications.^3,4^ SnS thus bears a huge potential for sustainable solar energy conversion, which is largely unexploited to date with only a handful of studies reporting SnS-based solar cells.^4–13,18–20^

To date, a range of fabrication methods have been used for the preparation of semiconducting SnS films.^14^ These include atomic layer deposition (ALD),^7^ chemical vapour deposition (CVD),^5,15^ thermal evaporation,^4,8^ sputtering,^16^ sulfurisation,^12^ spray pyrolysis,^3,17^ electrodeposition,^9^ successive ionic layer adsorption and reaction (SILAR)^18^ or chemical bath deposition (CBD).^19–21^ However, these techniques are, to a large extent, either vacuum-based or involve slow deposition rates, which is not favourable for low-cost and large-scale production. In this context, it is desirable to process SnS thin films from solution using coating or printing techniques compatible with high throughput manufacture.

In addition, the use of such solution-based fabrication techniques is particularly attractive as it enables the deposition of SnS in combination with organic semiconducting materials to form hybrid solar cells. The use of such hybrid structures affords greater design flexibility, allowing, for example, the optimization of key photovoltaic device parameters such as open circuit voltage and photocurrent. However, studies reporting the use of hybrid SnS–organic composite materials in solar cells have been limited to date.^10,11,18–20^ For example, in a report by Wang *et al.* ^10^ SnS nanoparticles were blended with a conjugated polymer and applied as absorber layer in hybrid solar cells. It was found that upon addition of the SnS nanoparticles the device performance improved compared to a pristine polymer solar cell, although, the power conversion efficiency (PCE) remained under 0.1%. SnS was also investigated as absorber in semiconductor sensitised solar cells.^11,18–20^ In most of these studies on SnS-based hybrid solar cells the open circuit voltages (*V*_OC_s) are increased compared to inorganic thin film SnS solar cells and PCEs between 0.01 and 1.3% are reached. One publication reports a PCE of 2.8%.^19^ However, none of them discloses significant photocurrent generation over the entire absorption range of SnS based on external quantum efficiency (EQE) spectra, while this would be a crucial prerequisite to exploit the full potential of SnS as PV-material in hybrid solar cells.

In this communication, we report a novel solution-processed route for the preparation of nanostructured SnS layers and demonstrate their suitability for efficient charge generation in hybrid photovoltaic devices.

Firstly, we consider the fabrication of the SnS films from solution. Full experimental details are provided in the ESI.† Briefly, a precursor solution containing SnCl_2_ and thioacetamide (TAA) in pyridine was deposited on a substrate (*via* spin coating) to form a precursor layer. The resulting precursor film was then converted into a SnS layer by thermal annealing in inert atmosphere. TAA decomposes upon heating of the precursor layer at around 105 °C,^22^ releasing reactive sulfur species, which in turn react with the Sn^2+^ ions to form the metal sulfide in a solid state reaction. The other reaction products are volatile and evaporate during the annealing process. X-ray diffraction patterns of as prepared films at annealing temperatures of 200, 250 and 300 °C are shown in Fig. 1A. It can be seen that in the samples annealed at 250 and 300 °C all the characteristic peaks of orthorhombic SnS (Herzenbergite, reference pattern: PDF 14-0620) are present in the diffraction patterns. In the sample prepared at 200 °C the peaks are comparably weak and broadened, which indicates that this temperature is too low to form a well-crystallised material. At 250 °C some additional peaks (marked with an asterisk) are visible and can be assigned to SnS_2_, which is formed as a secondary phase at this annealing temperature. Annealing at 300 °C leads to an almost complete elimination of this secondary phase. Only two very weak peaks, which may be due to SnS_2_ can be seen in the diffraction pattern of this sample. The narrow and sharp peaks in the diffraction pattern indicate that the formed material is highly crystalline and an estimation of the primary crystallite size using Scherrer equation revealed crystallite sizes of approx. 60 nm. Moreover, the disproportionally high 040 peak (full diffractogram is shown in Fig. S1 in the ESI†) indicates that the SnS sample crystallised preferentially along the (040) planes, as also already observed before.^23^

**Fig. 1:**
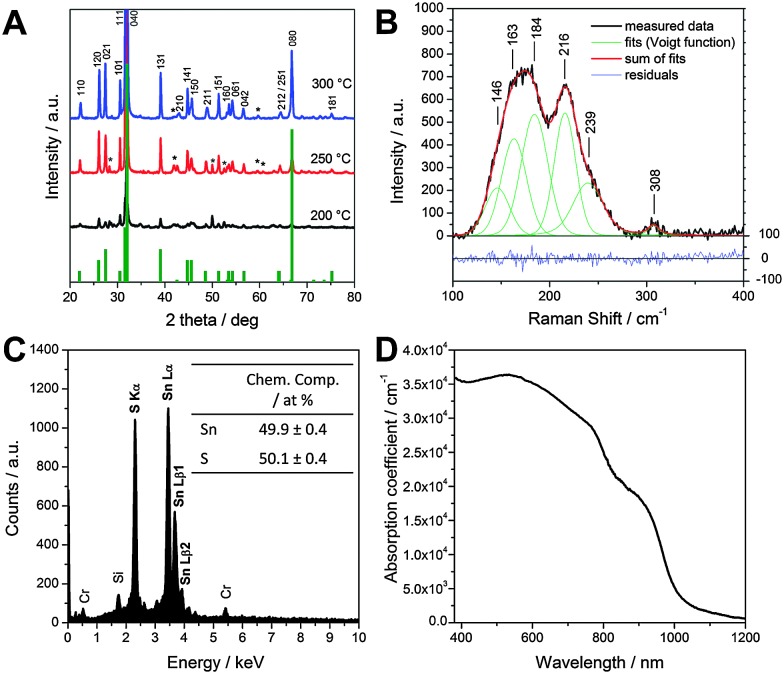
X-ray diffraction patterns of SnS layers prepared at 200, 250 and 300 °C (A), Raman spectrum (B), SEM-EDX spectrum (C) and absorption coefficient (D) of the SnS layer prepared at 300 °C.

To further investigate the SnS sample prepared at 300 °C, Raman spectra were recorded using a laser wavelength of 633 nm. The spectrum shown in Fig. 1B exhibits two broad Raman peaks. Fitting these peaks using Voigt function revealed five single peaks which can be assigned to the B_3g_ (146 cm^–1^), B_2g_ (163, 184 cm^–1^) and A_g_ (216, 239 cm^–1^) Raman modes of SnS and are consistent with literature data.^23–25^ The minor peak at 308 cm^–1^ is most likely due to traces of SnS_2_ in the sample.^24,26^

The analysis of the chemical composition of this sample using SEM-EDX analysis (see Fig. 1C) reveals an atomic ratio for Sn : S of 1 : 1, thereby supporting the formation of tin monosulfide as also revealed in XRD and Raman studies. We note that the peaks for Si and Cr in the EDX spectrum stem from the glass substrate and a thin Cr layer, deposited on the sample to prevent charging of the sample by the electron beam in the SEM, respectively.

Fig. 1D shows the characteristic UV-Vis spectrum of SnS films with a strong absorption over the whole visible range and a part of the near infrared region of the spectrum. We observe in Fig. 1D an absorption onset slightly under 1000 nm (corresponding to the direct band gap of approx. 1.3 eV) and a weaker absorption edge between 1100 and 1150 nm (matching with an indirect band gap of approx. 1.1 eV). The corresponding Tauc plots are presented in the ESI† (Fig. S2). Furthermore, it should be noted that the prepared SnS layers have high absorption coefficients up to 3.5 × 10^4^ cm^–1^, which is typical for SnS^4,8^ and also comparable to the more established solar absorber material CZTS.^27^

SEM images of the prepared SnS samples (Fig. 2A and B) reveal that the layers consist of a porous network of SnS nanoplates, which have a thickness of 60–80 nm and vary in diameter between a few 100 nm up to 1 μm (see also Fig. S3, ESI†). This porous nature of the SnS electrode provides a high surface area and therefore the potential for use in excitonic hybrid inorganic–organic solar cells. To assess the use of such SnS films in hybrid solar cells we first investigated the infiltration of the porous SnS nanoplate network with the organic conjugated polymer poly(3-hexylthiophene-2,5-diyl) (P3HT). The SEM images in Fig. 2C and D show that the pores and voids in the SnS layers are homogeneously filled with P3HT. The lighter regions in the SEM images correspond to SnS nanoplates covered by a P3HT film.

**Fig. 2:**
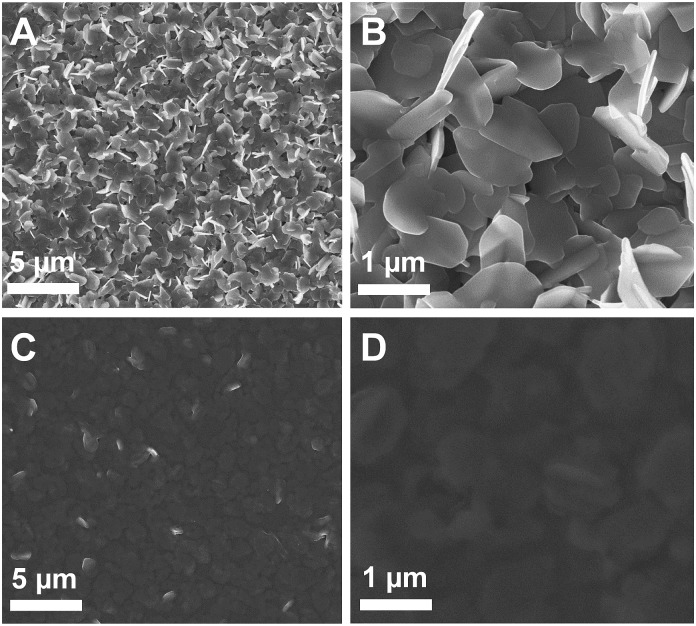
SEM images of the prepared layers: (A) overview image of the nanostructured SnS layer, (B) image showing the SnS nanoplates, (C, D) SnS layers shown in A and B coated with a P3HT layer.

Next, we studied the charge generation properties at the SnS–P3HT heterojunction using microsecond transient absorption spectroscopy. Fig. 3A shows the transient absorption spectra of planar-TiO_2_/SnS and planar-TiO_2_/SnS/P3HT films taken 10 μs after pulsed excitation at 510 nm. The shape of the transient absorption spectrum for the planar-TiO_2_/SnS/P3HT sample (black curve, Fig. 3A) resembles that reported for hole-polarons in P3HT.^28^ In contrast, TiO_2_/SnS (Fig. 3B) and TiO_2_/P3HT (Fig. S4, ESI†) samples showed no transient absorption signals within the time resolution of our transient absorption spectrometer (IRF ∼ 100 ns). We therefore conclude that the transient signal in the planar-TiO_2_/SnS/P3HT is due to charge separation across the SnS–P3HT heterojunction. We note that in the planar-TiO_2_/SnS/P3HT film hole polarons in P3HT are generated either by hole transfer from SnS to P3HT, or by exciton dissociation at the SnS/P3HT interface and subsequent electron transfer from P3HT to SnS. Both mechanisms are energetically favourable according to the energy levels of the materials involved (see Fig. S5, ESI†). The kinetics of the charge recombination reaction between the photogenerated electrons and holes were determined by monitoring the decay of the P3HT^+^ polaron signal at 1000 nm. Typical decay dynamics for the planar-TiO_2_/SnS/P3HT (black curve) and planar-TiO_2_/SnS samples are presented in Fig. 3B. As can be seen in the graph, the lifetime of the charges *t*_50%_ (defined by the time taken for 50% of the initial concentration of P3HT^+^ polarons to decay back to ground state) is ∼100 μs, which is comparable to lifetimes found in other metal sulfide–P3HT nanocomposites.^29^

**Fig. 3:**
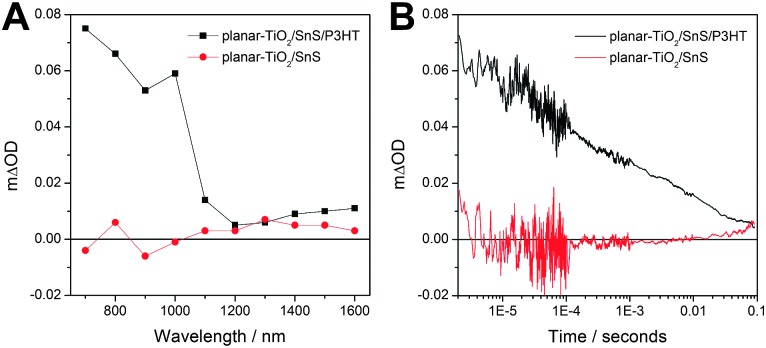
(A) Transient absorption spectra of a SnS as well as a SnS–P3HT layer on planar-TiO_2_ 10 μs after pulsed excitation at 510 nm and (B) transient absorption kinetics of the same samples recorded at 1000 nm following excitation at 510 nm. Corresponding steady-state absorption spectra of the investigated samples are shown in the ESI† in Fig. S6.

To address the suitability of SnS–P3HT films as a photoactive material, we fabricated hybrid SnS–P3HT solar cells, using the following inverted device architecture: glass/ITO/planar-TiO_2_/SnS/P3HT/MoO_3_/Ag. A detailed description of the used device fabrication procedure as well as their characterisation is provided in the ESI.† Fig. 4A shows current–voltage (*IV*) characteristics of a typical SnS–P3HT solar cell measured in the dark and under 100 mW cm^–2^ illumination. The solar cell exhibits a short circuit current (*I*_SC_) of 11.6 mA cm^–2^, a *V*_OC_ of 276 mV and a fill factor (FF) of 0.38, which leads to a PCE of 1.2%.

**Fig. 4:**
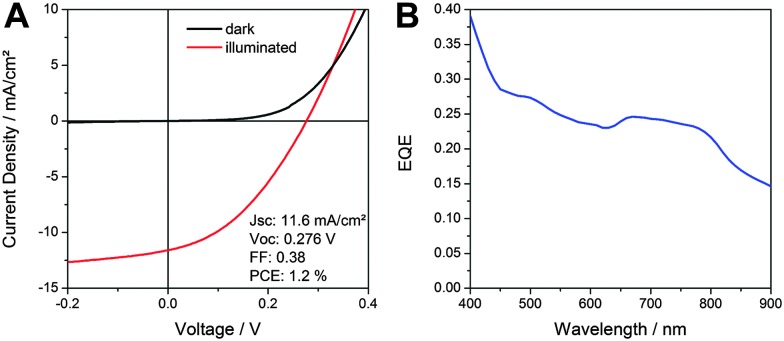
(A) *IV* curves in the dark and under 100 mW cm^–2^ illumination and (B) EQE spectrum of a nanostructured SnS–P3HT hybrid solar cell.

The short circuit current of the prepared solar cells is the highest presented so far for SnS-based inorganic–organic hybrid solar cells. The basis for a high short circuit current of this type of solar cells is the photocurrent generation over a broad range of the solar spectrum, as is revealed by the EQE spectrum in Fig. 4B. Already at a wavelength of 900 nm, where SnS is the only absorbing material, the EQE shows significant photocurrent generation, which increases to a value of approx. 0.4 at 400 nm. In view of the absorption spectrum of the SnS–P3HT absorber layer (Fig. S6, ESI†), we expect photocurrent generation also in the wavelength range between 900 and 1000 nm. The *V*_OC_ of the prepared hybrid solar cells is comparable to *V*_OC_-values of inorganic thin film SnS solar cells. Nevertheless, higher *V*_OC_s in thin film solar cells could be achieved by optimisation of the interlayers.^7,12^ In this sense, we are confident that the *V*_OC_ of hybrid SnS-based solar cells can also be increased by studying and optimising the hybrid interface, which opens up a new strategy for further research in the field of SnS-based solar cells.

In summary, we introduced a facile and rapid solution-based method to prepare SnS nanoplate networks, which can be infiltrated with an organic conjugated polymer resulting in nanostructured hybrid heterojunctions. Transient absorption spectroscopic measurements revealed that long-lived charges are generated in the SnS–P3HT layers upon illumination, highlighting their potential for solar cell applications. Prepared hybrid solar cells in inverted device architecture showed very promising short circuit currents based on absorption in a broad spectral range and for the first time a significant contribution of the SnS phase to charge generation in a hybrid solar cell is demonstrated. Improvement of the morphology of the hybrid layers to increase photocurrent and fill factor as well as strategies to overcome the limited photovoltage, which SnS-based solar cells have in common so far, in particular by means of optimising the hybrid interface, are topic of current research.

Financial support from the Austrian Science Fund (FWF) under the grant number J3515-N20 is gratefully acknowledged by TR. S.A.H. acknowledges financial support from the Engineering and Physical Sciences Research Council (EPSRC) through (EP/H040218/2) and (EP/K010298/1) projects and from the European Community’s Seventh Framework Programme (Nanomatcell, grant agreement number 308997).
