# Supplementary material for: Formation of porous SnS nanoplate networks from solution and their application in hybrid solar cells
Source: Chem Commun (Camb). 2015 May 27;51(50):10198–201. doi: 10.1039/c5cc03125g (PMC4479932; doi:10.1039/c5cc03125g)
Supplement: Supplementary file 1 [file CC-051-C5CC03125G-s001.pdf]

Electronic Supplementary Material (ESI) for ChemComm.
This journal is © The Royal Society of Chemistry 2015

Supplementary Information

to

Formation of porous SnS nanoplate networks from solution and their application in hybrid solar cells

T. Rath, L. Gury, I. Sánchez-Molina, L. Martínez and S. A. Haque

Experimental details:

Sample preparation:

The precursor solutions for the preparation of the SnS layers were prepared by dissolving 94.8 mg (0.5 mmol, 1 equiv.) $SnCl_2$ (98%) and 150.3 mg (2 mmol, 4 equiv.) thioacetamide (TAA, ≥99.0%) in 1 mL of pyridine (≥99%). The solutions were stirred for 5 min and filtered through a 0.45 µm PTFE syringe filter to give a clear colourless solution.

SnS layes were prepared by spin coating (1000 rpm) or dropcasting (for XRD and SEM-EDX samples) the precursor solution onto glass substrates, which were covered with a planar TiO_2 layer. Next, the precursor layers were heated to 200, 250 or 300 °C for 10 min in inert atmosphere, whereby the precursor layer is thermally converted into a nanostructured SnS layer.

Planar TiO_2 layers on glass or glass/ITO substrates were prepared by spin coating (6000 rpm) of a precursor solution (containing 70 µL titanium isopropoxide, 55 µL ethanolamine and 1 mL 2-methoxyethanol) and annealing the substrates for 1 h at 450 °C in ambient conditions.

Solar cell preparation:

Solar cells were prepared on glass substrates sputtered with ITO (Psiotec Ltd), which were cleaned in acetone and isopropanol in an ultrasonic bath. Next, a planar TiO_2 and also a SnS layer were prepared as described above. Afterwards, the nanostructured SnS layer was infiltrated with P3HT (Plexcore OS 2100) by spin coating a P3HT solution (17.25 mg in chlorobenzene) onto the SnS layer having a 30 s waiting time between applying the polymer solution and spinning (2000 rpm). The solar cell preparation was finished by thermal evaporation of a MoO_3 layer (10 nm) and an Ag layer (100 nm) on top of the SnS/P3HT layer.

Characterisation techniques:

X-ray diffraction patterns were measured on a PANalytical X'Pert Pro MRD diffractometer using Ni filtered Cu K_α radiation at 40 kV and 40 mA.

Raman spectroscopy was performed on a LabRAM Infinity spectrometer (Horiba) using a 633 nm He-Ne laser.

Steady-state absorption measurements were done with a Shimadzu 2600 spectrophotometer equipped with an ISR-2600Plus integrating sphere attachment.

SEM-EDX measurements were carried out on a JEOL 6400 scanning electron microscope operated at 20 kV.

Scanning electron microscopic images were acquired on a LEO 1525 Field Emission Scanning Electron Microscope operated at 10 kV using an In Lens detector. Samples were coated with chromium (5 nm) by sputtering prior to performing the SEM characterisations.

Micro-second transient absorption spectroscopy (µs-TAS) measurements were carried out by exciting the samples under a dynamic nitrogen atmosphere using a dye laser (Photon Technology International Inc. GL-301) pumped by a nitrogen laser (Photon Technology International Inc. GL-3300). A 100 W quartz halogen lamp (Bentham, IL 1) with a stabilized power supply (Bentham, 605) was used as a probe light source. The probe light passing through the sample was detected with a silicon photodiode (Hamamatsu Photonics, S1722-01). The signal from the photodiode was amplified before being passed through electronic band-pass filters (Costronics Electronics). The amplified signal was collected with a digital oscilloscope (Tektronics, DPO3012), which was synchronized with a trigger signal from the pump laser pulse from a photodiode (Thorlabs Inc., DET210).

IV characteristics of the prepared solar cells were measured using a Keithley 2400 source meter. The solar cells were illuminated (100 mW/cm^2) through shadow maks using a 150 W Xenon lamp (ScienceTech SS150W solar simulator) with an AM1.5 filter (ScienceTech). EQE spectra were measured using light from a quartz halogen lamp (Bentham IL1) filtered through a monochromator and a Keithley 2400 source meter.

Additional figures:

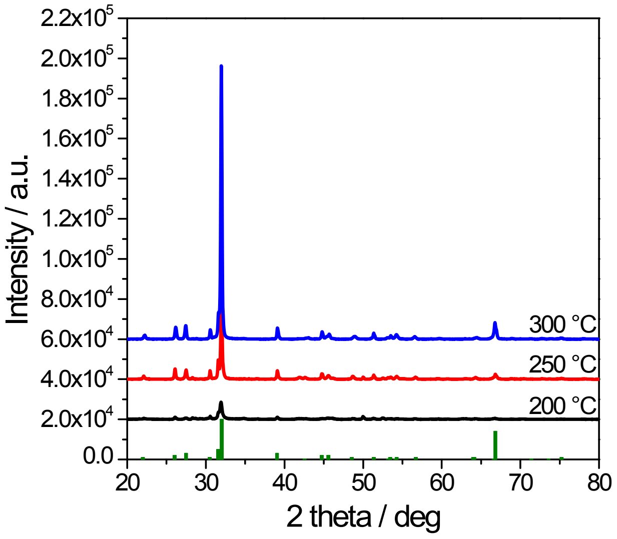

Fig. S1 X-ray diffraction patterns of SnS layers prepared at 200, 250 and 300 °C.

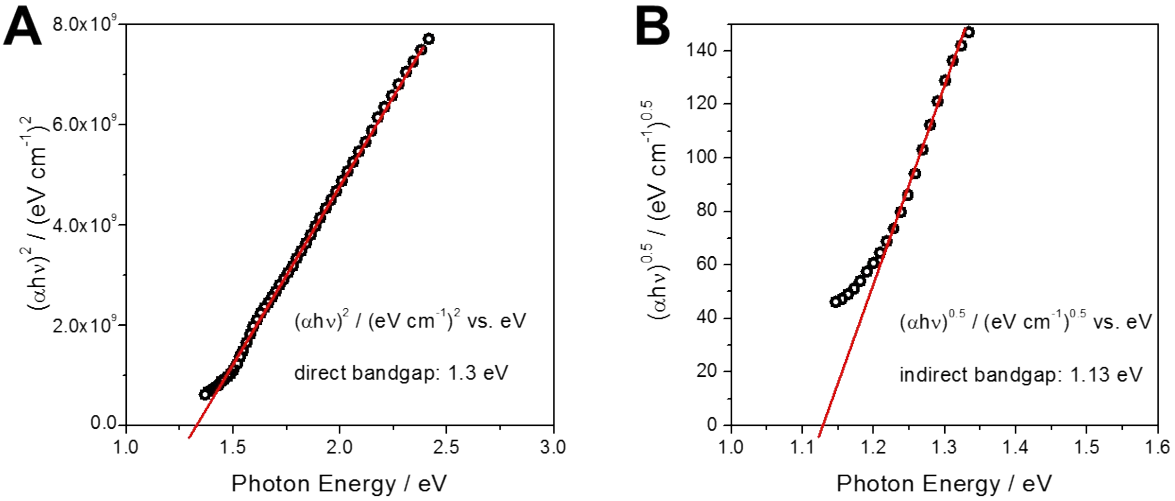

Fig. S2 Tauc plots to determine the direct (A) and indirect (B) band gap of the SnS sample prepared 300 °C.

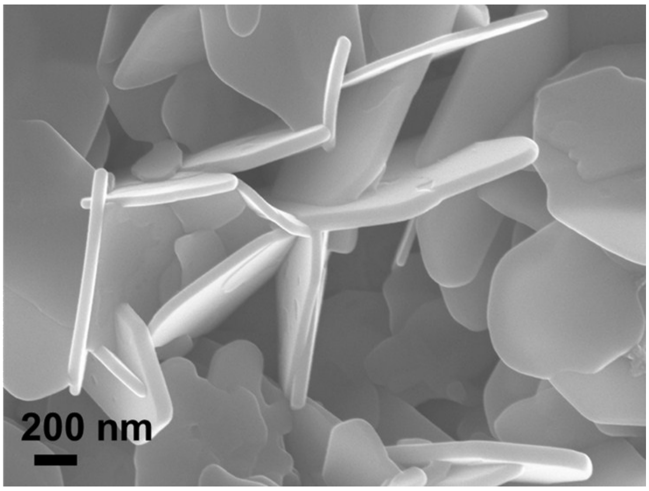

Fig. S3 SEM image (magnification 100 kx) of a prepared nanostructured SnS layer.

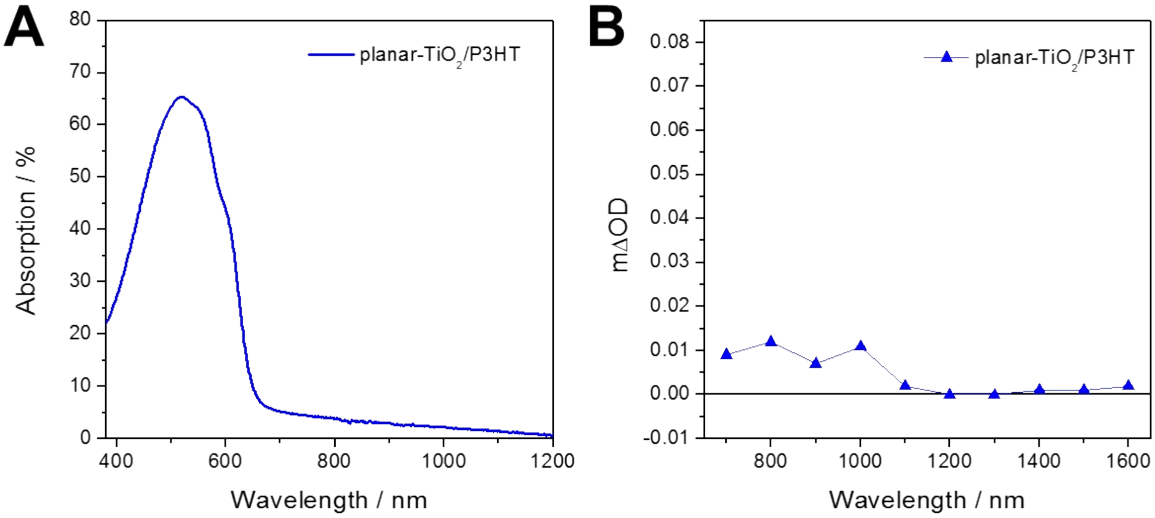

Fig. S4 (A) UV-Vis absorption spectrum of a P3HT layer on planar TiO_2 and (B) transient absorption spectrum of the same sample 10 µs after excitation at 510 nm.

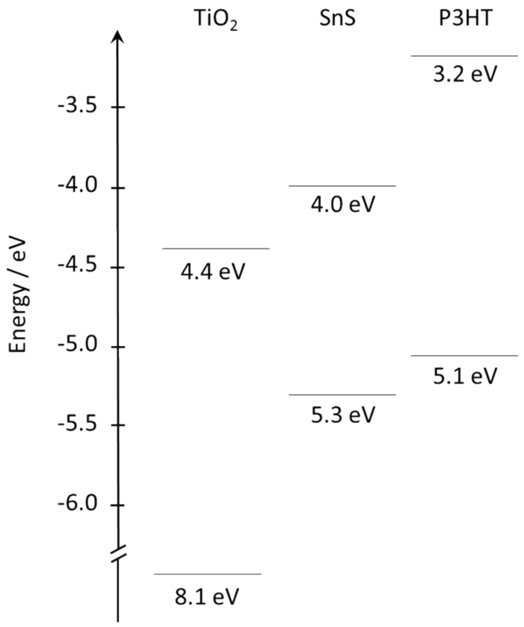

Fig. S5 Energy-level diagram of the involved materials according to values published in literature (TiO_2,[1] SnS,[2] P3HT[3]).

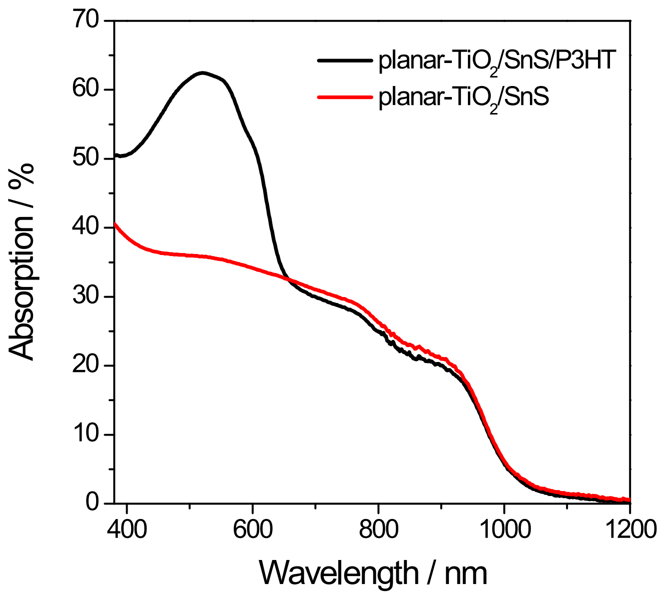

Fig. S6 UV-Vis absorption spectra of the samples investigated using transient absorption spectroscopy.

[1] T. Salim, Z. Yin, S. Sun, X. Huang, H. Zhang and Y. M. Lam, *ACS Appl. Mater. Interfaces*, 2011, **3**, 1063–1067.
[2] M. Sugiyama, K. T. R. Reddy, N. Revathi, Y. Shimamoto and Y. Murata, *Thin Solid Films*, 2011, **519**, 7429-7431.
[3] J. Y. Kim, K. Lee, N. E. Coates, D. Moses, T.-Q. Nguyen, M. Dante and A. J. Heeger, *Science*, 2007, **317**, 222-225.
